# Criteria-based audit to improve a district referral system in Malawi: A pilot study

**DOI:** 10.1186/1472-6963-8-190

**Published:** 2008-09-22

**Authors:** Eugene J Kongnyuy, Grace Mlava, Nynke van den Broek

**Affiliations:** 1 Child and Reproductive Health Group, Liverpool School of Tropical Medicine, Liverpool, UK; 2 Maternal and Newborn Health, The Health Foundation Consortium, Lilongwe, Malawi

## Abstract

**Background:**

To study the feasibility of using criteria-based audit to improve a district referral system.

**Methods:**

A criteria-based audit was used to assess the Salima District referral system in Malawi. A retrospective review of 60 obstetric emergencies referred from 12 health centres was conducted and compared with prior established standards for optimal referral of emergencies. Recommendations were made and implemented. Three months later, a re-audit was conducted (62 cases).

**Results:**

There were significant improvements in 4 out of 7 standards: adequate resuscitation before referral (33.3% vs 88.7%; p = 0.001); delay of less than 2 hours from the time the ambulance is called to when the ambulance brought the patient to the hospital (42.8% vs 88.3%; p = 0.014); clinician attends to patient within 30 minutes of arrival to hospital (30.8% vs 92.6%; p = 0.001) and feedback given to the referring health centres (1.7% vs 91.9%; p <0.001). The rest of the three standards showed a high level of attainment (>95%) in both the initial audit and the re-audit: referred patients accompanied by a referral form; ambulances are available at all times and the district hospital is informed through short-wave radio by the health centre when a patient is referred.

**Conclusion:**

Criteria-based audit can improve the ability of a district referral system to handle obstetric emergencies in countries with limited resources.

## Background

Malawi's Maternal Mortality Ratio, currently estimated at 1,100 per 100,000 live births, is one of the highest in the world [1]. Most of these deaths can be prevented by improving the availability, accessibility and utilization quality emergency obstetric care services [2]. Most health centres in Malawi cannot provide basic emergency obstetric care, which means that many obstetric emergencies are referred to district hospitals.

Salima District in Malawi has a population of 342,979 and 17,149 expected deliveries per year. The district has one hospital (Salima District Hospital) and 12 health centres. All these health centres have a functional short-wave radio linking them to the district hospital. There are three ambulances serving the whole district.

A baseline survey of the Salima District in 2006 revealed that 29% of deliveries were conducted in health facilities, the case fatality for emergency obstetric complications wais 1.4%, first visit antenatal coverage was 94.6% and postnatal coverage between 1 and 2 weeks was 11.5%, and the population-based Caesarean section rate was 2.8% [3]. The met need for emergency obstetric care (that is the proportion of Emergency Obstetric Complications treated in Emergency Obstetric Care facilities) was 6.4%. The baseline survey identified weaknesses in the referral system, quality of Emergency Obstetric Care and Women-friendly Care [3,4].

Salima District Hospital receives about 350 referrals for Emergency Obstetric Complications per year from the 12 health centres. Salima is the only Comprehensive Emergency Obstetric Care (CEmOC) facility in the district. There is no fully functioning Basic Emergency Obstetric Care (BEmOC) facility since none of the health centres provide all the 6 signal functions of a Basic Emergency Obstetric Care facility: 12 health centres provide parenteral antiobiotics, 12 provide parenteral oxytocics, 10 provide parenteral anticonvulsants, 4 provide manual removal of the placenta, 0 provides manual vacuum aspiration and 0 provides vacuum extraction [3].

The district referral system in Salima is financed by the Government and all referrals are provided free to all the citizens within the district. Salima District Hospital has 4 State-registered nurse-midwives, 17 enrolled nurse-midwives, 9 clinical officers, 6 medical assistants and 2 laboratory technicians. Salima District Hospital has 7 labour beds and 24 postnatal beds.

Third level delay (particularly during referrals between health centres and the district hospital) has been identified as a major contributing factor to maternal deaths in Salima. We sought to improve this referral system by conducting a criteria-based audit.

## Methods

This study was conducted in Salima District in Malawi. This is a small district with good roads linking all health centres to the district hospital. Salima District has a surface area of 2,196 km^2 ^and the population density is 156 per km^2^. There is 1 health centre within 5 km from the District Hospital and 8 health centres within 30 km and the furthest health centre 46 km from the District Hospital.

We followed the classic steps of a clinical audit cycle in this study [5]. The first step was the establishment of standards for the Salima District referral system. Standards were established by a Quality Improvement Team, which is a multidisciplinary team made up of the District Health Officer (a nurse-midwife), Safe Motherhood Coordinator (a nurse-midwife), midwives, nurses, clinical officers, anaesthetic technicians and laboratory technicians. The clinical officers form a cadre of health professional who receive 3 years of training in medicine and do most of the things doctors do in hospitals such as consultations and operations including Caesarean sections. There is no medical doctor in Salima District. In addition to the Quality Improvement Team, ambulance drivers were invited to take part in the meeting for the establishment of these standards. A total of 21 participants were present in this meeting. Prior to this study the Quality Improvement Team had a 2-day training on criterion-based audit and maternal death audit. The Safe Motherhood Coordinator is the Quality Improvement Team Leader and coordinates the activities of this group. All other members have the responsibility of participating in meetings and carrying out specifically assigned tasks such implementation of recommendations made during the audit process. Case fatality rate was defined as the proportion of women referred for Emergency Obstetric Complications who die.

The second step was to measure the current practice. Current practice was measured by a retrospective review of all cases of Obstetric Emergency Complications (obstetric haemorrhage, pre-eclampsia/eclampsia, prolonged/obstructed labour, retained placenta, puerperal sepsis, complications of abortion, ectopic pregnancy) referred between April and May 2007. None of the cases referred died before arrival. Sources of information included maternity admission register, discharge register, case notes and referral forms. The health care provider who called the ambulance indicated on the referral form the time the ambulance was called and the provider receiving the patient in the District Hospital indicated in this form at what time the patient was received. During the monthly supervisory visits the supervisor (a nurse-midwife) from the District Hospital discussed and monitored any feedback on referrals with the health centre staff. Data was collected using a tool specially designed for this purpose. The tool had information of patient characteristics and standards for the referral system. One member of the Quality Improvement Team was assigned to collect data on each referred over the specified period of time.

The third step was to compare current practice with the standards. During the fourth step, recommendations were made and implemented to fill the gaps identified between current practice and standards. The results of the initial audit were presented and recommendations disseminated during a quality improvement workshop that brought together representatives from the district hospital and all the 12 health centres. Recommendations were made by the Quality Improvement Team. Implementation of the recommendations was facilitated by the fact that the District Health Officer and Safe Motherhood Coordinator were members of the Quality Improvement Team. Their support promoted the allocation of resources for implementation.

Three months later, the audit was repeated (August to September 2007) to assess the progress made. The study was approved by the Reproductive Health Unit of Malawi Ministry of Health. Table 1 presents the recommendations made and implemented.

**Table 1:** Recommendations made and implemented

1. Two ambulances were stationed in two health centres to serve the other nearby health centres
2. Health care providers (from both the district hospital and health centres) trained on the resuscitation, particularly the management of hypovolemic shock
3. Health care providers briefed on the need to handle emergencies urgently and properly
4. Ambulance drivers briefed on the need to handle emergencies urgently
5. All necessary preparations made to receive and treat the patient when a health centre calls to inform the district hospital about an emergency referral
6. Midwives on duty allowed to call the clinicians and anaesthetic technicians directly thereby bypassing the bureaucracy of calling first the senior midwife in-charge of the maternity who will decide whether to call for additional staff.

It was estimated that a minimum sample size of 52 was required for a power of 90% to detect a 30% increase (that is from 50% to 80%) in standard attainment between the first and second audits with significance level of 5% (Table 2). In a pilot baseline assessment, third level delay involving the referral system was found in 50% (5/10) of referred cases.

**Table 2:** Attainment of standards in the first and second audits

**Standards**	**Initial audit** ** (n = 60)**	**Re-audit** ** (n = 62)**	**OR (95% CI)**	**p-value**
All referred patients come with a referral form filled by the referring facility	58/60 (96.7%)	62/62 (100.0%)	1.03 (0.61–1.77)	0.848
Ambulances are available at all times to transport referred patients	60/60 (100.0%)	62/62 (100.0%)	1.00 (0.59–1.70)	1.000
Health centre staff inform the district hospital through the short-wave radio when a patient is referred	60/60 (100.0%)	62/62 (100.0%)	1.00 (0.59–1.70)	1.000
Health centre staff receive feedback on all patients referred	1/60 (1.7%)	57/62 (91.9%)	55.16 (10.08–1138.11)	<0.001
All patients are adequately resuscitated before referral	20/60 (33.3%)	55/62 (88.7%)	2.66 (1.43–5.01)	0.001
A delay of less than 2 hours from the time an ambulance was called to when the ambulance brought the patient to the district hospital	23/55 (41.8%)	53/60 (88.3%)	2.11 (1.15–3.92)	0.014
All patients referred are attended to by a clinician within 30 minute of arrival in the district hospital	16/52 (30.8%)	50/54 (92.6%)	3.01 (1.53–6.03)	0.001

## Results

There were no significant differences in the characteristics of patients referred during the initial audit and the re-audit (Table 3): mean age (p = 0.205), median parity (p = 0.156), indications for referral (all > 0.05), proportion of deliveries by Caesarean section (p = 0.635), perinatal mortality (p = 0.380) and case fatality (p = 0.521).

**Table 3:** Characteristics of women referred from health centres to Salima District Hospital

**Standards**	**Initial audit (n = 60)**	**Re-audit (n = 62)**	**p-value**
Mean age in years (Standard Deviation)	25(6)	24(6)	0.205
Median parity (range)	3(1–10)	3(1–9)	0.156
Indications for referral			
Prolonged/obstructed labour (%)	60.0	58.1	0.927
Obstetric haemorrhage (%)	13.3	11.3	0.686
Puerperal sepsis (%)	8.3	6.5	0.616
Preeclampsia/eclampsia (%)	6.7	11.3	0.444
Complications of abortion (%)	10.0	6.5	0.425
Retained placenta (%)	1.7	3.2	0.808
Delivery by Caesarean section (%)	31.7	35.5	0.635
Perinatal mortality (%)	8.3	4.8	0.380
Case fatality rate (%)	5.0	3.2	0.521

There were significant improvements in 4 out of 7 standards of care: adequate resuscitation before referral (33.3% vs 88.7%; p = 0.001); delay of less than 2 hours from the time the ambulance is called to when the ambulance brought the patient to the hospital (42.8% vs 88.3%; p = 0.014); clinician attends to patient within 30 minutes of arrival to hospital (30.8% vs 92.6%; p = 0.001) and feedback given to referring the health centres (1.7% vs 91.9%, p <0.001). The rest of the three standards showed a high level of attainment (>95%) in both the initial audit and the re-audit: all patients referred come with a referral form filled by the referring health facility; ambulances are available at all times to transport referred patients and the district hospital is informed by the health centre through short-wave radio when a patient is referred. Table 3 presents the results of the audit.

## Discussion

This study describes a criteria-based audit of a district referral system and showed significant improvements in the referral system. Previous studies on criteria-based audit in developing countries have focused on improving the quality of emergency obstetric complications [6-8]. This study differs from previous studies because it highlights the importance of the referral system in the management of emergency obstetric complications.

The strategy of the World Health Organisation (Department of Making Pregnancy Safer) is to reduce maternal mortality by strengthening the health systems. Strengthening health systems to ensure that skilled attendants are available within a continuum of care, including emergency referrals, has a positive effect not only on maternal and neonatal health but also on a wide range of other services too.

We involved the health care providers, senior management and support staff right from the very first step of criteria-based audit. Traditionally standards are established by experts often without input from those who will implement those standards [8]. Involvement of senior management promoted the implementation of changes that required allocation of resources and involvement of all health care providers and support staff such drivers promoted successful implementation, ownership and sustainability.

Our study confirm findings from previous studies that criterion-based audit could be implemented in resource-poor countries using minimal resources [3]. Each workshop or meeting that brought people from all over the district cost about 150 US Dollars. This cost includes food and transport of participants to the District Hospital. There was no accommodation cost as all participants returned to their homes at the end of each day. The very process of criterion-based audit promoted the allocation of resources for maternity care at the district level so that no external funding was required for implementation of recommendations made during the audit process.

There are many reasons why criterion-based audit of the referral system succeeded in Salima District. First of all the District is relatively small and has well equipped district hospitals. Secondly there were functional ambulances available at the District Hospital. Furthermore, there were functional short-wave radios linking all health centres to the District Hospital. However the referral systems needed a bit of re-organisation to handle emergencies urgently and properly and the hospital staff including ambulance drivers needed some refresher training on Emergency Obstetric Care. Hence the re-organisation of the referral system and training of health care providers led to the success.

It is not certain whether the immediate gains from criterion-based audit of the referral system in Salima District will be sustained. The involvement of the senior management of the District Hospital in the audit process promoted re-allocation of resources and there are hopes that their involvement will also promote sustainability. There are plans to continue follow up and assess the referral system using criterion-based audit over a longer period of time.

## Conclusion

In conclusion, criteria-based audit can improve the ability of a district referral system to handle obstetric emergencies in countries with limited resources. Future research should control for confounding factors (by using a control district for example) and assess effectiveness of criterion-based audit in terms of process and outcome indicators.

## Competing interests

The authors declare that they have no competing interests.

## Authors' contributions

EJK conceived the topic, designed the study, collected and analysed the data, interpreted the findings, prepared and finalised all versions of the manuscript. GM assisted in data collection. NVDB reviewed the manuscript for intellectual content.

## Pre-publication history

The pre-publication history for this paper can be accessed here:


